# Editorial: Immune-related adverse Events (irAEs) of biopharmaceuticals

**DOI:** 10.3389/falgy.2023.1214914

**Published:** 2023-05-24

**Authors:** Alexander Batista-Duharte, Salvador F. Aliño

**Affiliations:** ^1^ Immunology and Allergy Group (GC01), Maimonides Biomedical Research Institute of Cordoba (IMIBIC), University of Cordoba, Reina Sofia University Hospital, Cordoba, Spain; ^2^ Farmacogenetics and Gene Therapy Group, Instituto de Investigación Sanitaria La Fe, Valencia, Spain; ^3^ Gene Therapy and Pharmacogenomics Group, Department of Pharmacology, Faculty of Medicine, University of Valencia, Valencia, Spain

**Keywords:** immune-related adverse events (irAEs), proteins, nucleic acids, polysaccharides, cell-based therapy, delivery systems, vaccines, antibodies

**Editorial on the Research Topic** Immune-related adverse events (irAEs) of biopharmaceuticals

Biopharmaceuticals are a class of medicines derived from living cells or semi-synthesized from biological sources and are used to treat a variety of diseases, including cancer, autoimmune disorders, and infectious diseases. While COVID-19 vaccines have had a major impact on the biopharmaceutical industry in recent years due to the pandemic, monoclonal antibodies (mAbs) as a group have been the leading biopharmaceuticals in terms of approvals and sales (1).

Although biopharmaceuticals have been highly effective in the treatment of many diseases, they can also cause immune-related adverse events (irAEs). irAEs occur when the biopharmaceutical activates the immune system, primarily causing immune dysregulation and inflammatory/autoimmune effects. The severity and frequency of irAEs vary depending on the drug, treatment regimen, and the patient's individual conditions (2). In this Research Topic, there are six published articles; two original research, two reviews, one study protocol, and one case report. Five articles focused on irAEs associated with immune checkpoint inhibitors (ICIs)-based treatment and one article reviewed the toxicity caused by different biological immunotherapies used in COVID-19.

Immune checkpoints are regulatory proteins on immune cells that control the duration and intensity of the immune response to prevent autoimmunity. The use of ICIs has become a rapidly developing field, as a monotherapy or in combination with conventional therapies (3) and vaccines (4). In this Research Topic, Nakagomi et al. described the clinical course of four patients with ICI-related myositis overlapping with myocarditis of 583 patients treated with either a PD-1 or PD-L1 inhibitor alone or with a PD-1/CTLA-4 inhibitors combination. They focused on changes in myositis and myocarditis activity based on temporal changes in troponin and creatine kinase (CK) levels in serum, in response to immunosuppressive therapy. In all patients, CK levels normalized within a month of starting immunosuppressants, but troponin levels increased after treatment and it took more than two months to normalize. A systematic review was also conduced and found fourteen case reports of ICI-related myositis with myocarditis, which had a similar pattern to their study. The authors pointed out that this discordance could be explained because myocarditis is more resistant to immunosuppressive treatment than myositis and therefore a careful long-term follow-up with troponin is required in these patients.

A case report of acute mastitis associated with reactive cutaneous capillary endothelial proliferation after camrelizumab treatment was described by Wu et al. as a new immune-related adverse event. Camrelizumab is a humanized anti-PD-1 mAbs that acts as an ICI. The authors describe inflammatory changes and “mulberry-like” reactive cutaneous capillary endothelial proliferation (RCCEP) on the patient's left breast skin. RCCEP is a known irAE associated with camrelizumab treatment (5). However, in this case, mammary lesions were found inside the glands, in addition to RCCEP, being considered by the authors a new irAE. After stopping treatment, the patient's mastitis gradually improved. The description of this case can serve as a reference for other similar cases that may occur during treatment with camrelizumab or other ICIs.

A systematic review of case reports of opportunistic infections by *Pneumocystis jirovecii* pneumonia (PJP) associated with ICIs was showed by Xia et al. The authors identified 677 reports of PJP associated with ICIs treatment, of which 44.3% had a fatal outcome. Nivolumab, pembrolizumab (Anti-PD-1 mAbs), ipilimumab (Anti-CTLA-4 mAbs), atezolizumab, durvalumab (anti-PD-L1 mAbs), and nivolumab/ipilimumab showed significant associations compared to other drugs in FAERS database, especially in males aged >65 years. However, after excluding several confounding factors PD-1 inhibitors emerged with a strong disproportionality signal compared to PD-L1/CTLA-4 inhibitors and targeted therapy. The authors also point out that PJP may be unmasked in cancer patients during immune reconstitution induced by ICIs. This is the first large-scale pharmacovigilance study to investigate the association of PJP and ICIs by combining FAERS data mining and literature review.

Cutaneous toxicities are regarded as the earliest and most common irAE associated with ICIs therapy. Muhaj et al. offered an excellent review of mucocutaneous adverse events (irCAEs) related to ICIs. They describe the more frequent reactions including vitiligo, lichenoid dermatitis, psoriasiform dermatitis, and other less common, including bullous dermatoses, neutrophilic dermatoses, and autoimmune dermato-rheumatologic diseases. In general, these reactions can greatly impact the quality of life of the patients and their proper management is pivotal to prevent interruptions or suspension of life-saving immunotherapy. However, the authors highlight that many irCAEs such as vitiligo, flares of psoriasis, and possibly alopecia indicate a positive therapeutic response. For this reason, most irCAEs can be managed without discontinuation of ICIs.

In a protocol study presented on our Research Topic, Les et al. showed a protocol of a multicenter, prospective, observational cohort study to evaluate the diagnostic performance of an autoantibody panel consisting of antinuclear antibodies, rheumatoid factor, and antineutrophil cytoplasmic antibodies, to predict the occurrence of irAEs in patients treated with ICIs. This study protocol is the first phase of a project, to address an unmet need in the identification of reliable and validated biomarkers to predict the onset of irAEs in patients treated with ICIs.

Finally, a timely review by Baracaldo-Santamaría et al. provided a comprehensive understanding of the safety of several biopharmaceuticals currently recommended for COVID-19 treatment. They updated reported irAEs associated with anti-interleukin 6 receptor alpha (IL-6R*α*) mAbs, such as tocilizumab and sarilumab; siltuximab a blocker of human IL-6, Sotrovimab an anti-SARS-CoV-2 mAbs, and alternative drugs such as anakinra and canakinumab (both IL-1 receptor antagonist), Itolizumab, an anti-CD6 humanized mAb and baricitinib, a Janus kinase (JAK) inhibitors. The authors propose that patients with severe COVID-19 that present serious irAEs associated with IL-6 pathway inhibitors should be alternatively managed with baricitinib to manage the excessive immune response seen in these patients.

The papers collected in this Research Topic illustrate some of the many facets of irAEs associated with the most common biopharmaceutics that are being used currently. The best understanding and management of these reactions is a rapidly developing field that will allow the design of more effective and safer products for the treatment of complex diseases such as cancer, autoimmune disorders, allergies, and various types of infections.
